# Burnout, compassion for others and fear of compassion: a quantitative study in Iranian nurses

**DOI:** 10.47626/2237-6089-2020-0082

**Published:** 2021-10-22

**Authors:** Sajad Khanjani, Yousef Asmari Bardezard, Aliakbar Foroughi, Fayegh Yousefi

**Affiliations:** 1 Behavioral and Cognitive Science Research Center Department of Health, Rescue and Treatment of Police Force Tehran Iran Behavioral and Cognitive Science Research Center, Department of Health, Rescue and Treatment of Police Force, Tehran, Iran.; 2 University of Social Welfare and Rehabilitation Sciences Tehran Iran University of Social Welfare and Rehabilitation Sciences, Tehran, Iran.; 3 Department of Clinical Psychology Kermanshah University of Medical Sciences Kermanshah Iran Department of Clinical Psychology, Kermanshah University of Medical Sciences, Kermanshah, Iran.; 4 Department of Psychiatry Kurdistan University of Medical Sciences Sanandaj Iran Department of Psychiatry, Kurdistan University of Medical Sciences, Sanandaj, Iran.

**Keywords:** Burnout, compassion for others, fear of compassion, nurses

## Abstract

**Introduction:**

Occupational burnout in nurses is a barrier to job promotion and achievement of job-related goals, resulting in reduced hospital performance.

**Objective:**

Considering the importance of the role of compassion in occupational burnout among nurses, this cross-sectional study aimed to explore the burnout levels and their relationships with compassion for others and fear of compassion.

**Methods:**

This descriptive correlation study was carried out with 216 nurses who were selected using a multistage cluster sampling method and asked to respond to the Maslach Burnout Inventory, a compassion for others scale and Fears of Compassion Scales (FCS), which includes three subscales; fears of compassion for self, compassion for others, and compassion from others.

**Results:**

The results showed that burnout had a negative correlation with compassion for others, but had positive correlations with fear of compassion for others, fear of compassion from others, and fear of self-compassion. Moreover, the results of regression analysis showed that 21% of burnout variance was explained by compassion for others and 29% of its variance was explained by fears of compassion scales.

**Conclusion:**

Based on the findings, compassion can be considered as a protective factor in preventing burnout among nurses.

## Introduction

Healthcare workers are constantly exposed to stressors due to complex care and patient care services, which put them at risk of occupational burnout.^1^ Among healthcare workers, nurses reported the highest prevalence of burnout.^2^ Burnout syndrome is the constant exposure to work-related stress that is associated with poor working conditions and reduced job enjoyment and performance.^3^ The term burnout was first used in 1974 by Herbert J. Freunderberger in the United States to describe a range of symptoms, including decreased mood, lack of motivation to work, and mental and physical fatigue.^4^ In 1980, investigating health professionals, Maslach and Jackson defined burnout as “physical and emotional fatigue that leads to a decrease or loss of motivation to work, which also leads to feelings of failure at work.”^5^ In 1982, they developed a scale for assessing burnout that had three components: depersonalization, decreased personal performance, and emotional exhaustion.^5^ In 2019, the World Health Organization (WHO) described burnout as “chronic workplace stress that is not properly managed,” defining it as a syndrome in the 11th edition of the International Classification of Diseases (ICD-11), with three indications: 1) feelings of energy depletion or exhaustion; 2) increased mental distance, or feelings of negativism or cynicism related to one’s job; and 3) reduced professional efficacy. The International Classification of Diseases also identifies job burnout as a workplace-specific phenomenon that should not be used to describe similar experiences in other areas of life.^6^ A meta-analysis reported a 25% prevalence of burnout in Iranian nurses between 2000 and 2017.^7^ Another review and meta-analysis study showed that the global prevalence of burnout in nurses is 11.23%.^8^

Compassion is an important variable in care services. The English word “compassion” comes from Latin and Greek “pati” and “pathein” (meaning suffering) and “com” (with). So compassion means “suffering” with someone else.^9^ Compassion can be defined in different ways. For example, the Dalai Lama defined compassion as “openness to the suffering from others, with a commitment to alleviate it.”^10^ In recent years, the term “compassionate care” has been introduced because of the importance of compassion in health care settings.^11,12^ On the other hand, other terms such as “compassion fatigue” have also been introduced.^13^

Compassion can be directed to oneself^14^ or to others.^15^ Researchers have shown that sympathetic pity and concern about others through altruistic and voluntary acts are associated with psychological and physical benefits.^16,17^ Compassion for others is associated with better relationships, mood improvement,^18^ and improved psychological well-being.^19^ Greater compassion for spouses is associated with a lower risk of mortality.^20^ Beaumont et al. found that higher levels of compassion were associated with lower levels of burnout and compassion-related fatigue in student counselors and student cognitive behavioral psychotherapists.^21^ However, another study found no significant relationship between compassion for others and burnout in student midwives.^22^

On the other hand, research by Gilbert has shown that some people are afraid of expressing compassion.^23^ In Gilbert’s view, this fear can be in the form of fear of self-compassion, fear of compassion for others, and fear of compassion from others.^23^ Studies have shown that fear of compassion is associated with fear of happiness and symptoms of psychopathology such as anxiety, stress, and depression.^24-27^ So, perhaps one of the reasons for the exhaustion of the caregiver is the fear of compassion. Furthermore, in recent years, it has been opposed to the ‘compassion fatigue’ term.^28,29^ Singer and Klimecki suggested that rather than compassion fatigue, we should think about empathy fatigue.^28^ Based on Singer and Klimecki’s work, Dowling suggests increasing the level of compassion through mindfulness meditation.^29^

Although compassion is an important factor in mental health and well-being, the results of research into the relation between compassion for others and burnout are contradictory. There was a significant negative relationship between compassion for others and burnout in cognitive-behavioral therapists and counseling students. However, in another study, with midwifery students, no relationship between these two variables was found. On the other hand, research shows that fear of compassion is related to psychopathology. Therefore, considering the importance of compassion in nursing and the lack of adequate research into the relationship between compassion for others and fear of compassion with burnout in nurses, the aim of this study was to investigate in Iranian nurses the relationship with occupational burnout of compassion for others and fear of compassion.

## Method

### Participants

Using the Gipower program with the specified values (effect size F^2^ = 0.15, α err prob = 0.01, power (1-β err prob) = 0.99, number of predictors = 9), a sample size of 220 people was estimated for this study. Four questionnaires were incomplete and only 216 were included in the analysis. Inclusion criteria include having at least a Bachelor’s degree in nursing, a minimum of 2 years’ work experience, and 1 year working full time in an intensive care unit (ICU) or coronary care unit (CCU).

This was a cross-sectional, descriptive-analytical study in which relationships between burnout, compassion for others, and fears of compassion scales were examined. The study was carried out between June 2016 and February 2017. The sample consisted of 216 nurses (114 men, 52.8% and 102 women, 47.2%) who were selected with a multistage cluster sampling method.

### Measures

#### Demographic questionnaire

Demographic data on the participants were obtained using a self-administrated questionnaire.

#### Compassion for others scale

This is a self-report measure that was developed by Pommier in 2010. It consists of 24 items encompassing three contradictory components (each component has two opposing elements) including kindness-indifference, common humanity-separation, and mindfulness-isolation. The participants scored the items on a five-point Likert response scale ranging from almost never (0) to almost always (4). The overall Cronbach’s alpha for compassion for others was 0.90 and Cronbach’s alphas for sub-scales of kindness, indifference, common humanity, separation, mindfulness, and isolation were reported as 0.77, 0.68, 0.70, 0.64, 0.67 and 0.67 respectively. Compassion for others was positively and significantly correlated with the social communication scale, the empathic concern subscale of Davis’ Interpersonal Reactivity, the personal distress subscale of Davis’ Interpersonal Reactivity, and Mehrabian’s Questionnaire of Empathic Tendency, and had a negative and significant correlation with the Reactivity Index (r = -0.15), (r = 0.59).^15^ The scale’s construct validity and reliability have been confirmed in research with an Iranian sample.^30^

#### The fears of compassion scales

This scale was developed by Gilbert et al. in 2011. It consists of three sub-scales for measuring fears of compassion: 1) fear of feeling or expressing compassion for others; 2) fear of receiving compassion from others; and 3) fear of self-compassion. The scale is scored based on a five-point Likert scale ranging from totally disagree (0) to totally agree (4). Cronbach’s alphas for subscales of fear of compassion for others, fear of receiving compassion from others, and the fear of self-compassion in nurses were reported as 0.70, 0.80, and 0.83 respectively. Cronbach’s alphas for therapists were reported as 0.75, 0.85, and 0.86 respectively.^23^

#### Burnout Inventory

The Burnout Inventory is a tool commonly used for measuring burnout. This tool was developed by Maslach. It consists of 22 items for measuring emotional exhaustion, depersonalization, and reduced sense of personal accomplishment within the framework of professional activity. This tool is especially useful for measuring and preventing burnout in professional groups such as nurses and teachers, etc. Maslach and Jackson tested the reliability of this instrument using Cronbach’s alpha. Alphas were reported as 0.90 for emotional exhaustion, 0.79 for depersonalization, and 0.71 for reduced sense of personal accomplishment.^31^ In Iran, the three factor model of this inventory was confirmed and alphas were reported as 0.76 for emotional exhaustion, 0.60 for depersonalization and 0.70 for reduced sense of personal accomplishment.^32^

## Data analysis

Data were cleaned and screened. Missing data were >5% of the dataset. Thus, list-wise deletion with no imputation of data was used in the present analyses. The assumptions of normality were checked and skew was not evident in the subscales or total scale score in this sample. The Cronbach’s alphas for burnout, compassion for others, fear of compassion for others, fear of compassion from others, and fear of self-compassion were 0.80, 0.78, 0.70, 0.74, and 0.85, respectively, indicating satisfactory internal consistency of these measures. The data collected were analyzed using, the *t* test for independent samples, analysis of variance (ANOVA), Pearson’s correlation coefficient, and multiple regression analysis, enter method, with SPSS software, version 20.

## Results

The mean burnout score for the sample was 62.84 (standard deviation [SD] = 11.26). In this study, participants were aged 23 to 55 years old and mean age was 31.40±6.80 years. Table 1 presents associations between demographic data, burnout, compassion for others, fear of compassion for others, fear of compassion from others, and fear of self-compassion. In addition, the *t* test for independent samples was conducted to investigate gender, education, and marital status-based difference in burnout, compassion for others, fear of compassion for others, fear of compassion from others, and fear of self-compassion (as dependent variables), with gender, education and marital status used as an independent variable in the analysis. There was only a significant gender-based difference in compassion for others (t = -2.84; p = 0.005). Moreover, the results of ANOVA showed that there were no significant work shift-based differences in burnout, compassion for others, fear of compassion for others, fear of compassion from others, or fear of self-compassion (p ≥ 0.05).

**Table 1:** Associations between demographic data, burnout, compassion for others, fear of compassion for others, fear of compassion from others, and fear of self-compassion

Variable	Burnout		Compassion for others		FOC for others		FOC from others		Fear of self-compassion	

	M (SD)	p-value	M (SD)	p-value	M (SD)	P value	M (SD)	p-value	M (SD)	p-value
Gender		0.37		0.005		0.69		0.14		0.07
Male	63.5 (11.2)		76.3 (11.8)		20.1 (5.4)		23.8 (6.3)		27.1 (9.1)	
Female	62.1 (11.3)		80.7 (10.9)		19.7 (6.0)		22.5 (6.2)		24.7 (9.3)	
										
Marital status		0.20		0.54		0.17		0.25		0.32
Single	63.8 (11.3)		77.8 (12.6)		20.5 (6.7)		23.7 (6.3)		26.5 (9.9)	
Married	61.8 (11.1)		78.8 (10.6)		19.3 (6.4)		22.7 (6.4)		25.3 (8.3)	
										
Education		0.96		0.51		0.67		0.83		0.39
BSc in nursing	62.8 (11/4)		78.0 (12.2)		20.9 (5.8)		23.3 (6.2)		25.5 (9.4)	
MSc in nursing	62.9 (10.9)		79.1 (10.1)		19.7 (7.2)		23.1 (6.0)		26.7 (9.1)	
										
Working shift		0.19		0.53		0.57		0.67		0.76
Morning	65.9 (12.3)		77.1 (12.2)		20.5 (6.2)		23.5(7.6)		25.8 (9.6)	
Afternoon	61.5 (10.6)		79.2 (11.2)		20.7 (5.7)		23.7(6.4)		26.7 (9.2)	
Night	62.5 (10.3)		80.5 (11.2)		19.7 (5.6)		22.3 (5.6)		24.3 (7.6)	
All shifts	61.1 (10.7)		77.5 (11.5)		18.7 (8.0)		22.8 (4.4)		26.1 (9.6)	

FOC = fear of compassion; M = mean; SD = standard deviation.

Table 2 shows that burnout had positive correlations with fear of compassion for others (r = 0.50; p ≤ 0.001), fear of compassion from others (r = 0.31; p ≤ 0.001), and fear of self-compassion (r = -0.37; p ≤ 0.001), whereas fear of compassion for others had a negative correlation (r = -0.47; p ≤ 0.001).

**Table 2:** Means, standard deviations, and correlation coefficient scores for burnout, compassion for others, fear of compassion for others, fear of compassion from others, and fear of self-compassion

Variable	M±SD	1	2	3	4	5
1. Burnout	62.84±11.26	-	-0.47*	0.50*	0.31*	0.37*
2. Compassion for others	78.39±11.61		-	-0.36*	-0.22*	-0.32*
3. Fear of compassion for others	19.96±6.30			-	0.33*	0.37*
4. Fear of compassion from others	23.26±6.48				-	0.61*
5. Fear of self-compassion	25.92±9.25					-

M = mean; SD = standard deviation.

* p < 0.01.

Table 3 shows that 21 percent of the total variance in burnout is characterized by compassion for others. The F ratio suggests that the regression model of burnout based on compassion for others is meaningful (F = 9.09; p ≤ 0.001). This means that the regression model is statistically significant. The results of regression coefficients also show that the t values obtained for common humanity (t = -2.22; p = 0.027) and separation (t = 3.10; p = 0.002) are both meaningful. This means that only two compassion for others subscales significantly predict burnout.

**Table 3:** Regression analysis of burnout based on compassion for others

Predictor variables	R	R^2^	F	Sig of F	B	SE.B	β (95%CI)	p-value	Collinearity statistics	

									Tolerance	VIF
	0.45	0.21	9.09	0.001						
Constant					57.05	5.68		0.001		
Kindness					-0.13	0.26	-0.04	0.617	0.60	1.66
Indifference					0.39	0.32	0.10	0.222	0.56	1.77
Common humanity					-0.59	0.27	-0.17	0.027*	0.68	1.46
Separation					0.75	0.24	0.27	0.002*	0.51	1.98
Mindfulness					-0.10	0.28	-0.03	0.720	0.60	1.65
Disengagement					0.29	0.29	0.08	0.307	0.65	1.55

95%CI = 95% confidence interval; SE.B = standard error for the unstandardized beta; Sig = significance; VIF = variance inflation factor.

* p < 0.01.

Table 4 shows that 29 percent of the total variance in burnout is characterized by fears of compassion scales. The F ratio suggests that the regression model of burnout based on fear of compassion for others, fear of compassion from others, and fear of self-compassion is meaningful (F = 29.57; p ≤ 0.001). This means that the regression model is statistically significant. The results of regression coefficients also show that the t values obtained from fear of compassion for others (t = 6.60; p ≤ 0.001) and fear of self-compassion (t = 2.40; p = 0.017) are meaningful. This means that only two of the fear of compassion scales significantly predict burnout.

**Table 4:** Regression analysis of burnout based on fear of compassion scales

Predictor variables	R	R^2^	F	Sig of F	B	SE.B	β (95%CI)	p-value	Collinearity statistics	

									Tolerance	VIF
	0.54	0.29	29.57	0.001						
Constant					61.09	6.31		0.001		
Fear of compassion for others					0.74	0.09	0.41	0.001*	0.84	1.19
Fear of compassion from others					0.11	0.13	0.06	0.391	0.62	1.62
Fear of self-compassion					0.22	0.11	0.18	0.017^†^	0.60	1.67

95%CI = 95% confidence interval; SE.B = standard error for the unstandardized beta; Sig = significance; VIF = variance inflation factor.

* p < 0.01; ^†^ p < 0.05.

## Discussion

This study aimed to investigate the relationship between burnout and the compassion for others scale and fears of compassion scales. The correlation coefficient results showed that the overall compassion for others score was negatively correlated with burnout in nurses. The results of the regression analysis also revealed that 21% of the total variance of burnout is negatively predicted by compassion for others. The results therefore indicate that nurses with high scores for compassion for others experience less burnout. This is consistent with the research by Beaumont et al. with student counselors and student cognitive-behavioral psychotherapists,^21^ but is not consistent with research by Beaumont et al. with midwifery students.^22^ These findings can be explained in several ways. Based on the stress-buffering model of social support, people who have compassion for others are more likely to be supported by others and are therefore less reactive to stress. Compassion for others is associated with a greater likelihood of accepting social support from others and thus greater protection against disease.^33,34^ Accepting compassion or support from others creates a buffer against numerous mental diseases that can be associated with psychological well-being.^33^ Thus, people who are more compassionate to others receive more compassion from others. In other words, due to the relationship between compassion for oneself and others,^35^ people who are more compassionate to others are more likely to be kinder to themselves and have less self-criticism. For this reason, they may be less likely to burn out. According to the Dalai Lama: “If you want others to be happy practice compassion. If you want to be happy, practice compassion”^10^ there is a relation between compassion for oneself and for others.

In addition, the results demonstrated that fears of compassion scales (fear of compassion for others, fear of receiving compassion from others, and fear of self-compassion) were positively correlated with burnout. Also, the results of regression analysis showed that 29% of the total variance of burnout is positively predicted by fears of compassion scales. The results showed that people with higher fears of compassion scores report higher levels of burnout, which is consistent with earlier studies that reported the relationship between fear of compassion and depression, anxiety, stress, and psychopathology.^23-27^ The relationship between fear of self-compassion and burnout can be explained in that people with fear of self-compassion have internalized a high level of self-criticism. Neff considered self-kindness against self-judgment as an aspect of self-compassion.^14,35^ People who have higher self-judgment tend to experience higher levels of stress, anxiety, and depression,^36^ which can lead to further fatigue and exhaustion.

Research shows that some people see a relationship between positive emotions and expectation of negative consequences.^23^ Unfortunately, this makes many people fear happiness. The emotional experience of compassion is an important positive emotion, especially for self-soothing, and its absence can prevent people from becoming more self-compassionate.^23^ On the other hand, those who have fear of self-compassion are more likely to have fear of compassion from others. In community and clinical samples, fear of self-compassion and of compassion from others were associated with psychological distress and lower levels of well-being,^37^ which in turn can be associated with burnout in nurses. People who fear receiving compassion from others have a fear of positive emotions such as affection and care, which are considered as fundamental barriers to secure attachment and positive emotion about themselves and others.^23^ Attempting to explain the relationship between fear of compassion for others and burnout, we can say compassion for others is beneficial to the individual, especially when in stressful situations compassion is given by others, and it can provide a buffer against stress. It has also been shown that compassion for others can be associated with prosocial behaviors and even physical and psychological well-being,^16,17,19^ which is a result of increased activation of the vagus nerve and the autonomic parasympathetic nervous system.^38,39^ Nevertheless, fear of compassion for others can be accompanied by withdrawal from others and lack of support for others, which can itself leave a person unprotected in stressful situations, and in such situations an individual’s energy reduces faster, which can lead to psychological and physical problems and eventually burnout.

There are some research limitations that can be considered for future studies. First, this study employed a cross-sectional, correlational design that cannot determine causality. Thus, experimental designs could be used in future studies. Second, the research data were obtained by self-report tools, the responses to which could be biased with a chance of socially appropriate responses. Third, in this study, other variables such as compassion fatigue that could contribute to burnout were not investigated. Further studies could also investigate the relationship between these variables.

In response to a lack of research into the variables of compassion for others and fear of compassion in the context of burnout in nurses, this study was conducted in order to investigate the relationship between burnout and compassion for others and fears of compassion scales. The results of this study proved that compassion for others could be associated with lower levels of burnout in nurses and somehow be positive for nurses’ psychological and occupational health. On the other hand, fear of compassion scores were associated with high levels of burnout in nurses. Compassion can therefore be considered a protective factor in preventing burnout among nurses.
